# Impact of Shredded Rubber Waste (SRW) on the Range of Elastic Work of Road Construction Mixtures Containing Industrial Waste Bound with a Binder

**DOI:** 10.3390/ma15238503

**Published:** 2022-11-29

**Authors:** Konrad Walotek, Joanna Bzówka, Adrian Ciołczyk

**Affiliations:** Department of Geotechnic and Road Construction, Faculty of Civil Engineering, The Silesian University of Technology, 44-100 Gliwice, Poland

**Keywords:** rubber waste, shredded rubber waste, composite mix, road construction, deformability, mining waste, industrial waste, circular economy, DIC

## Abstract

The paper presents the results of research on a composite mixture intended for use in road construction. The purpose of developing the mixture is to be able to use large amounts of industrial waste to produce building material. The waste used are coal slate from the mining industry, shredded rubber waste from used passenger car tires and fly ash from power plants. The mixture (SRFC) consists of unburnt coal-mining slate (S), shredded rubber waste (R), fly ash (F) and cement(C). A test under cyclic loading conditions was carried out on samples prepared from the SRFC mixture, in which the global deformations and local strains caused on the samples were measured. A measurement system using digital image correlation was used for the research. On the basis of the conducted research, it was found that the content of shredded rubber waste significantly influences the deformability of the tested mixtures and allows for the extension of the scope of elastic deformation in which the tested samples work.

## 1. Introduction

Management of rubber waste is becoming a growing problem worldwide. Year by year, this waste is increasing as a result of the development of the automotive industry. It is estimated that the annual production of automobile tires reaches about 1.4 billion units and a comparable amount becomes waste [1,2]. This waste is not considered as hazardous waste due to its lack of toxic properties, resistance to mould, moisture and bacterial attack. However, combined with resistance to biodegradation, organic solvents and the irreversible characteristics of the changes occurring in vulcanizates make them a major environmental burden [3]. As a result of the irreversible changes occurring during the vulcanization of fresh rubber compounds, direct recycling is not possible, so other methods of reuse should be sought, such as [4,5]:

- Energy recycling
- Product recycling
- Raw material recycling
- Material recycling

Product recycling and material recycling are most commonly used in the construction industry. For the former, car tires are used for [6,7,8,9,10,11,12,13,14,15,16]:

- Construction of road embankments
- Improving slope stability
- Retaining wall construction
- Construction of drainage and cut-off layers
- Construction of membranes and drainage layers
- Construction of road culverts

Material recycling is mostly based on mechanical or cryogenic [1] shredding of car tires and segregation of the resulting waste into textile, metal and rubber waste. Shredded rubber waste in the construction industry is used, among others [1,17,18]:

- For the modification of bituminous binders and mineral-asphalt mixtures
- As an additive to reinforce the subsoil
- Concrete additive

When **Shredded Rubber Waste SRW** is used as an additive to concrete, it results in a reduction in bulk density, compressive strength, water absorption, and an increase in frost resistance, deformability and fatigue life. Additionally, in their work, the authors noted the effect of **SRW** in moving away from the brittle fracture mechanics characteristic of concrete towards a less violent form of failure [19,20,21,22,23,24,25,26,27,28,29,30]. However, for concrete as a structural material, its stiffness characterized by the course of the stress–strain relationship is the most important characteristic, since the design of concrete structures assumes minimal strain at very high stresses. Hence, the use of **SRW** in these cases may adversely affect the behaviour of the structure.

As a result of the significant development of coal mining, the Upper Silesian Coal Region is rich in mining waste accompanying hard coal. These wastes are generated during preparatory work and mining and processing operations. According to the Road and Bridge Research Institute [31], more than 1.5 billion tonnes of post-coal waste is deposited in Upper Silesia and approximately 37 million tonnes are extracted annually [32]. Such a large amount of material requires finding a way to dispose of it or use it in industry.

Mining waste consists of various types of rocky and non-rocky soils and may include [31,33,34,35,36,37]:

- Clay slates
- Mudstones
- Carbonaceous slates
- Sandstones
- Pebbles
- Mules
- Siderite
- Sphero-siderites
- Coal crumbs

As the petrographic composition of the barren rock varies so much, it will show large differences in physical and mechanical parameters depending on its place of origin. In addition, as the barren rock is only subjected to natural erosion processes after extraction, it is very sensitive to the effects of water and frost, which cause degradation of the aggregate grain size. As a result of these changes, the waste rock may decrease its load-bearing properties by reducing the skeleton consisting of coarser aggregate fractions and may begin to exhibit heaving characteristics as a result of a significant accumulation of clay-silt fractions [31,38,39,40,41,42].

The above characteristics mean that any use of waste rock in the construction industry requires a number of laboratory tests, particularly long-term tests in which sensitivity to water and frost is tested. This causes considerable reluctance to use this waste among contractors, despite its attractive price and very high availability in Upper Silesia.

Post-coal waste is mainly used for:

- Embankment construction [31,43,44,45,46].
- Macro levelling of human-degraded land [47,48].
- The formation of hydraulic structures [38,39,49,50].
- As an additive in the manufacture of masonry elements and cements [47,51].

This paper presents the results of a study of an SRFC composite consisting of three waste materials (Unburnt coal-mining slate “S”, shredded rubber waste “R”, fly ash “F”) bound by a hydraulic binder “C” (cement CEM I 42.5). The aim of developing the composite is to produce a useful building material consisting mainly of industrial waste. Ultimately, the composite is to be used in the construction of road construction layers and in earthworks. The basic component of the mix is unburnt coal-mining slates obtained as waste from hard coal mining in Upper Silesia. This aggregate is to constitute the load-bearing backbone of the whole mix, but it is characterised by high sensitivity to water, which causes degradation of the aggregate grain size. In order to limit the influence of water on the aggregate, the addition of shredded rubber waste was used, which, among other uses, is supposed to isolate the aggregate grains from the influence of water. Additionally, the use of **SRW** is expected to improve the fatigue life of the composite, which is very important when using the material for the construction of road surface layers. Fly ash used in the mixture is to grain it in order to obtain the most closed structure. Cement serves in this case as a binder gives the proper compression strength to the whole.

This paper presents the results of a study of the deformability of a composite with varying **SRW** content, since it will determine its applicability. In particular, the focus is on the values of strain and elastic deformation. For this purpose, cyclic tests were proposed, based on the CBR cyclic test methodology [52]. The deformability tests were performed using the DIC ARAMIS 3D system, which allows precise determination the strain and deformations occurring on the surface of the tested specimen and free elaboration the test results [53,54].

## 2. Materials and Methods

### 2.1. Materials

**Figure 1:**
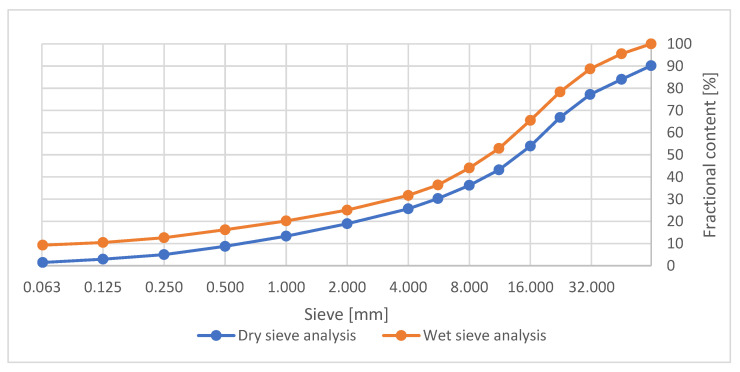
Grain size curve of unburnt coal-mining slate.

For the preparation of the composite mix, unburnt coal-mining slate from the process of decarburization of waste rock at the Haldex plant (Poland) was used. The aggregate used naturally has a grain size of 0/63 mm. Figure 1 shows the grain size curve of the aggregate.

**Figure 2:**
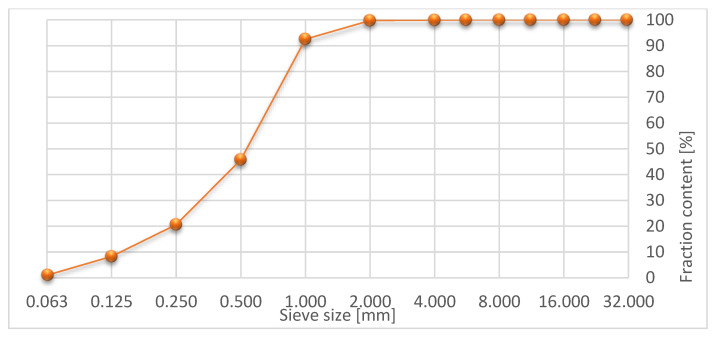
Grain size curve of shredded rubber waste.

The shredded rubber waste came from the process of mechanical shredding of used passenger car tires. **SRW** was used in the form of 0/2 mm grid size. The grain size curve is shown in Figure 2.

**Figure 3:**
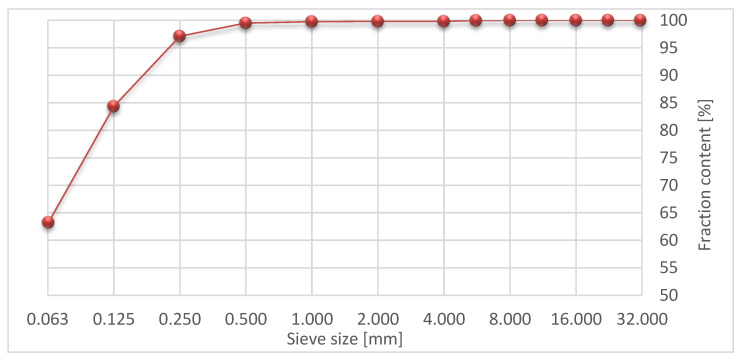
Grain size curve of silica fly ash.

Silica fly ash from coal combustion in a power plant was used. Figure 3 shows its grain size.

### 2.2. Preparation of the Composite Mix

The presented mixture was prepared on the basis of soil stabilization (according to WT-5). That is, the addition of shredded rubber waste, fly ash and cement were determined as a percentage of the dry mass of the aggregate, and the whole mixture was mixed and compacted at the optimum moisture content determined by the Proctor II method in accordance with Table 1.

Due to the low volumetric density value of the shredded rubber waste, a suitable mixing procedure was required to obtain a homogeneous mixture.

The procedure for preparing the mixture is as follows:

- Drying of all ingredients to constant weight at 105 °C
- Weighing the contents of the individual ingredients into separate containers
- Mixing together of shredded rubber waste, fly ash and CEM I 42.5 cement
- Adding to the mixture of shredded rubber waste, fly ash and cement about half of the water content needed to achieve optimum moisture content and mixing until a homogeneous mass is obtained
- Then the above mixture is added to the unburnt coal-mining slate with constant stirring. Mixing is carried out until the mixture becomes homogeneous, but not for longer than 5 min in order not to break up the aggregate grains too much.
- Once a homogeneous mass is obtained, the remaining water content is gradually added and mixed until homogeneous (no more than 2 min).

### 2.3. Preparation of Samples

The test specimens were compacted in 80 × 80 mm cylindrical moulds using a light hand tamping machine (2.5 kg). Compaction was carried out in two layers of 15 tamping strokes per layer. Due to the dimensions of the mould, the aggregate used for sample preparation was sifted to 0/16 mm grain size. Table 2 shows the recipes of the prepared mixtures.

**Figure 4:**
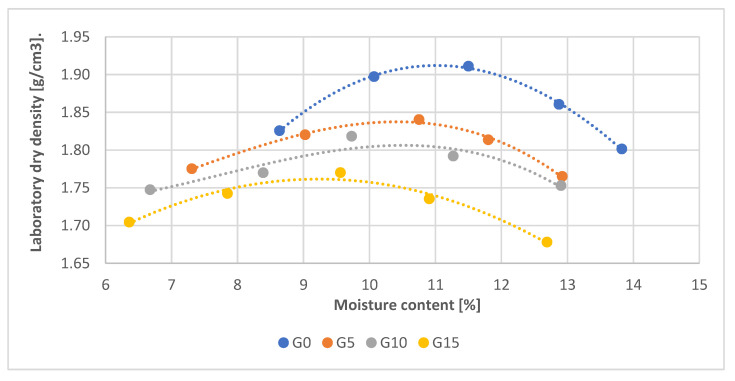
Plot of the dependence of the laboratory dry density on moisture content.

The samples were compacted at optimum moisture content (Figure 4). Five samples were prepared from each recipe for testing.

### 2.4. Research Plan

**Figure 5:**
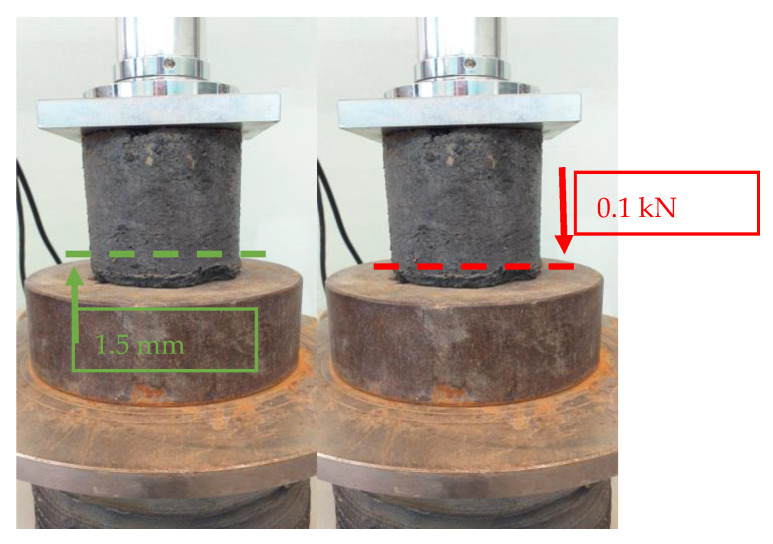
Scheme of a single cycle of loading and unloading the specimen (own photo).

Cyclic loading tests were conducted on the prepared samples after 28 days of curing. The test procedure was modelled on the cyclic CBR test [31]. The procedure for performing the test was as follows:

- At the end of the treatment period the sample is placed in the testing machine
- We preload the specimen with a force of approximately 1.5 kN in order to align the contact surfaces of the specimen and to compensate for play in the compression fixtures.
- The specimen shall be subjected to 20 cycles of loading and unloading or until failure at a speed of 1.27 mm/min.
- During cyclic loading and unloading of the specimen, the compressive force and deformation of the specimen must be continuously monitored.
- Each of the loading and unloading cycles should consist of (Figure 5):
  ○ Forcing a deformation of 1.5 mm on the specimen
  ○ Relieving the specimen to a compressive force not exceeding 0.1 kN

**Figure 6:**
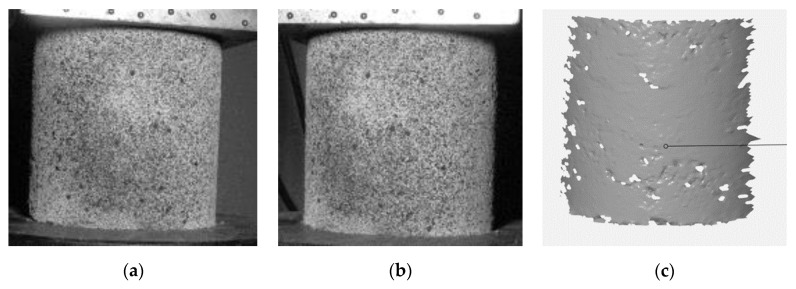
Measurement results using ARAMIS 3D system (**a**) image from the left camera; (**b**) image from the right camera; (**c**) 3D model built on the basis of images from cameras [own photo].

The test measured the global deformation of the specimen, read as absolute displacement values of the moving shelf of the testing machine. The measurement was performed continuously throughout the test. In addition, an ARAMIS 3D DIC system (HUB 150 mm) was used to precisely determine the deformations and strains occurring on the specimen surface. For this purpose, a stochastic pattern was made on the surface of each test specimen (Figure 6).

**Figure 7:**
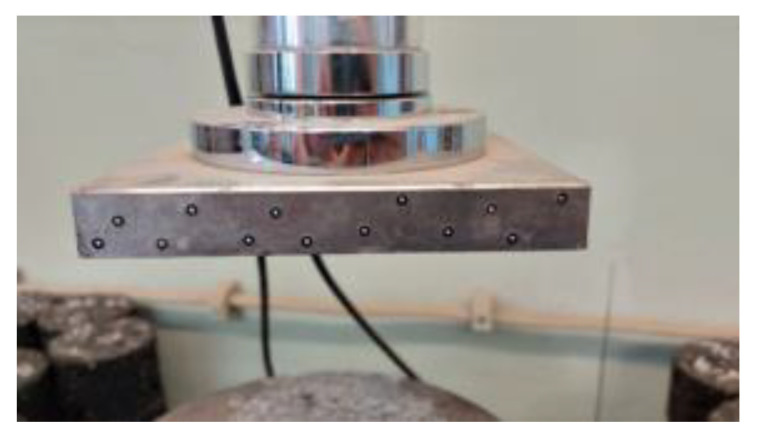
Measuring points on the testing machine attachment (own photo).

In addition to specimen preparation, the testing machine was also equipped with special measuring points to control the displacement of the compression fixtures during the test. The location of the test points is shown in Figure 7.

**Figure 8:**
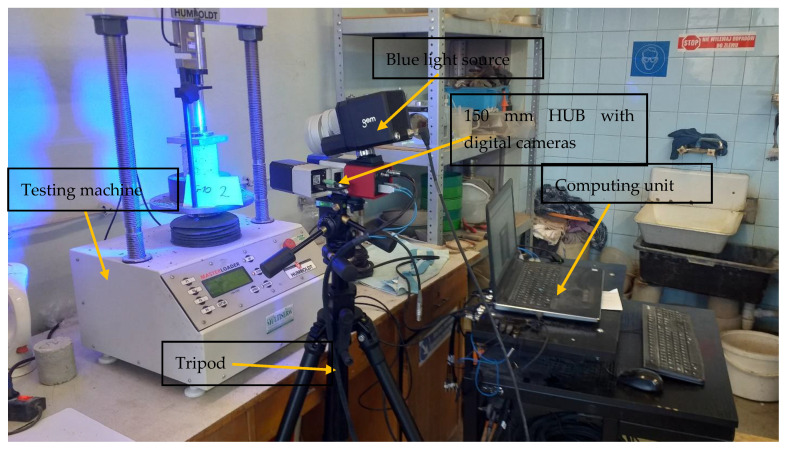
Stand prepared for cyclic loading test [own photo].

Figure 8 shows the complete stand prepared for the test. The bench consisted of the following components:

- Computing unit (laptop computer) with installed GOM Correlate Professional 2020 software
- Tripod
- A Humboldt HM-3000 testing machine with a maximum load of 50 kN and a maximum speed of 70 mm/min. The testing machine was coupled with a calculating unit to read and record the forces and deformations acting on the specimen
- 150 mm HUB with two digital cameras, 350 mm focal length and 150 × 120 × 90 mm research area
- Sources of blue light illuminating the test object

**Figure 9:**
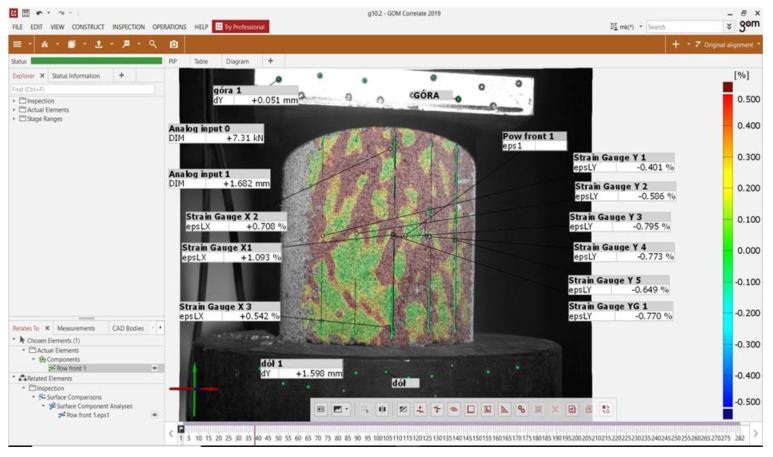
Measurement interface (own photo).

In order to measure the deformation and strain of the test specimens using the DIC system, measurement points were marked on the surface of the specimens. Figure 9 shows the view of the specimens from the GOM Correlate program along with the specified measurement areas.

The interface included the following measurement points:

- Analog input 0—a combination of a computational unit and a test press used to provide a constant reading of the compressive force acting on the test specimen. Due to the noisy nature of the readings from the test press, the values were corrected using a temporary binomial filter with a value of 2 (the value of “2” corresponds to the number of adjacent frames of the recording that are taken into account when averaging the force readings).
- Analog input 1—connection between the computing unit and the testing press, used for constant reading of the displacement value realized by the press. Due to the high “noise” of the readout values, the reading had the following control function
- Bottom 1—Fixed points marked on the lower (movable) platen of the testing machine. They were used for precise measurement of vertical deformation of the specimen, forced by the testing press
- Top 1—Fixed points marked on the top (stationary) shelf of the testing machine. They were used to control the deflection of the articulated specimen compression attachment
- Pow.Front.1—a surface element built on the basis of stochastic painting of specimens, used for measurement of main strains occurring on the specimen surface, according to the colour legend shown on the right side of the figure (range of principal deformations from +0.5% to −0.5%)
- Strain Gauge Xi (i = 1, 2, 3)—virtual strain gauges applied on the front surface of the specimen allowing for determination of transverse strain caused by compression of the specimen at various heights of the tested object. The strain gauges had a length of about 55 mm, the choice of such a large measuring system resulting from the attempt to measure, as precisely as possible, the global transverse strain of the specimen. The strain gauges were distributed as follows:
  ○ Strain Gauge X1—transverse strain gauge located at the centre of specimen height to measure maximum transverse strain of the specimen
  ○ Strain Gauge X2—a transverse strain gauge located in the upper visible area of the specimen to measure transverse strain at the top of the compression fixture.
  ○ Strain Gauge X3—a transverse strain gauge located in the lower visible portion of the specimen to measure transverse strain at the lower compression attachment
- Strain Gauge Yi, and ϵ <1;5>—virtual strain gauges applied to the front surface of the specimen to determine the vertical strain due to compression of the specimen. These strain gauges had a length of about 70 mm. The choice of such a large measuring base resulted from the attempt to measure the global vertical strain of the specimen as accurately as possible. The strain gauges were positioned from left to right of the visible side of the specimen. The number of strain gauges depended on the quality of the gradient painted on the surface of the specimen and ranged from 4 to 6. The values of vertical strain were then averaged for the whole specimen.

**Figure 10:**
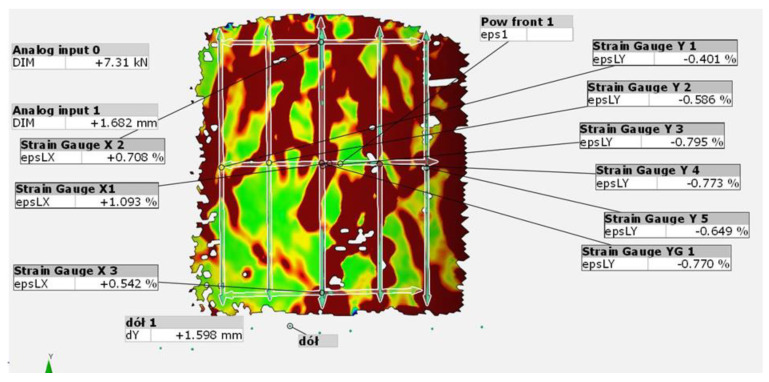
Example of setting up the strain gauge grid on the specimen surface.

Figure 10 shows an example of a strain gauge grid applied to the specimen surface.

## 3. Results

### 3.1. Course of Deformation and Strains during the Test

**Figure 11:**
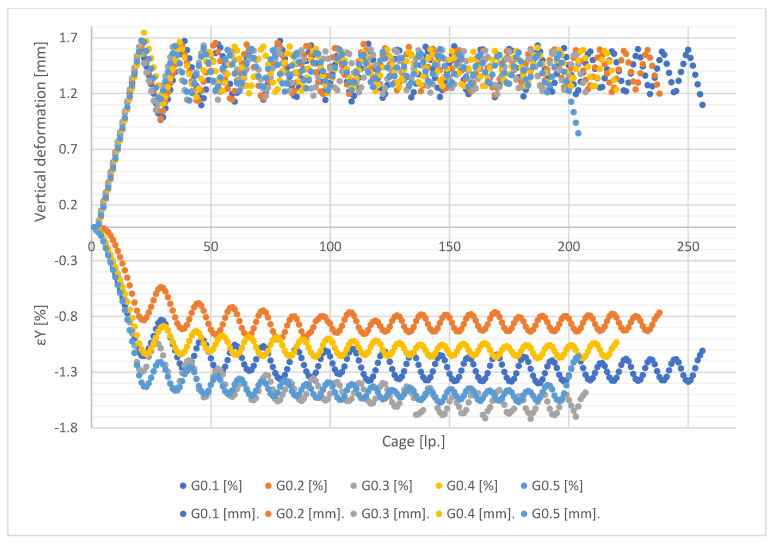
Results of measurement of deformation and vertical strain for G0 series specimens.

**Figure 12:**
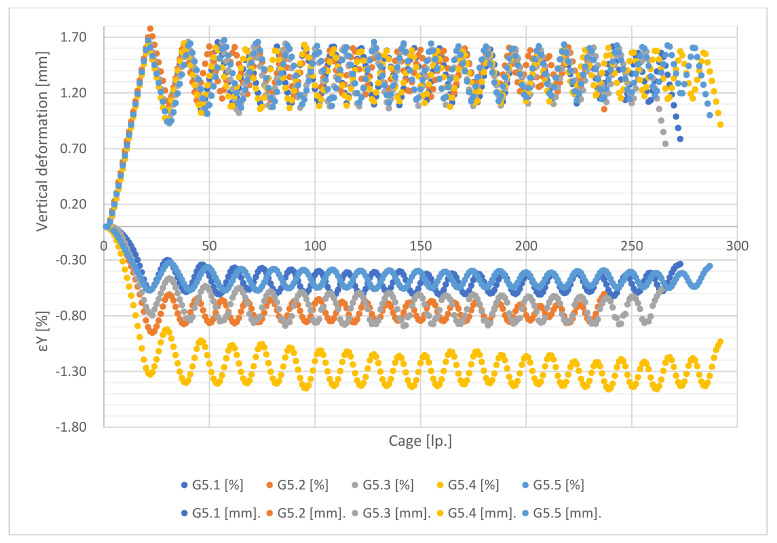
Results of measurements of deformations and vertical strains for G5 series specimens.

**Figure 13:**
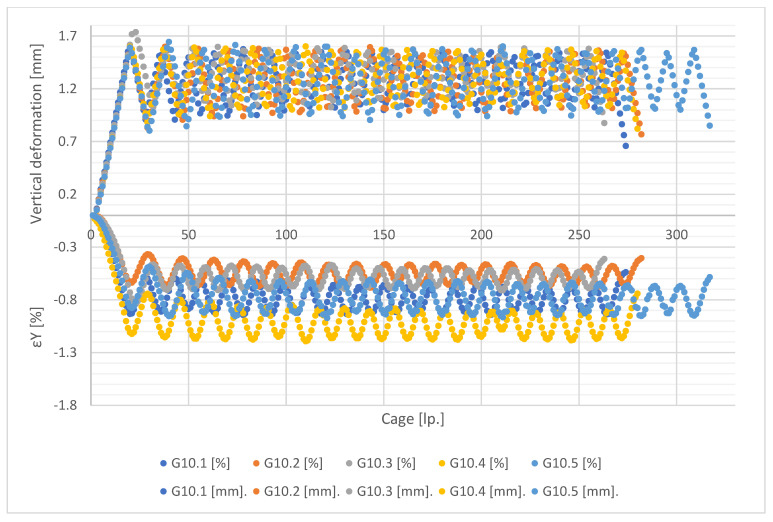
Results of measurement of deformation and vertical strain for G10 series specimens.

**Figure 14:**
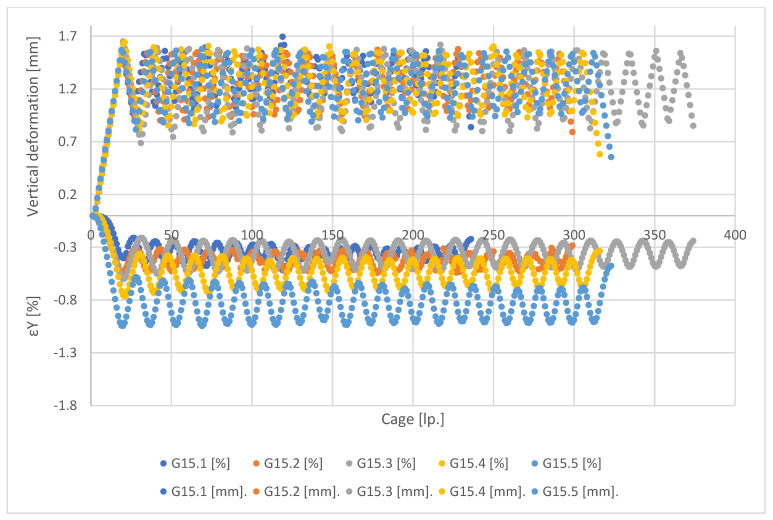
Results of measurement of deformation and vertical strain for G15 series specimens.

Figure 11, Figure 12, Figure 13 and Figure 14 show the global forced vertical deformation data on the specimen and the vertical strains on the specimen surface, during the cyclic loading test. The global deformation values, expressed in millimetres, are shown on the positive part of the vertical axis, while the strain values read from the virtual strain gauges, expressed in percentage, are shown on the negative part of the vertical axis. The horizontal axis contains the frame numbering of the recording made with the ARAMIS 3D DIC. Each graph shows the results of the test performed for five specimens from the same mixture.

Analysing the values of global deformations forced on the specimen, it can be observed that as the content of shredded rubber waste increases, the amplitude between the value of maximum forcing set in the test (about 1.5 mm) and the value obtained at the minimum assumed specimen load (0.1 kN) increases. As a result, the lowest values of amplitudes are observed for specimens of G0 series, while the highest values are for G15 specimen. A tendency is also visible for the magnitude of the amplitude to decrease with each successive loading cycle for all tested mixtures. This trend is higher in the first four loading cycles, with a decrease in the subsequent cycles. However, again the SRW content affects the magnitude of the change in this amplitude. As their content increases, the differences between cycles become smaller and smaller. In the case of G0 series samples, we observe the highest tendency for the amplitudes to decrease in successive test cycles, while in the case of G15 series samples this tendency is the lowest. Samples of G5 and G10 series present similar values of amplitude decrease.

In the case of the analysis of the strain values measured on the surface of the tested specimens, it can be firstly noticed that they present much higher scatter of the obtained results, for individual specimens, in comparison with the global deformation measurements. These scatterings decrease with an increase of SRW content. Table 3 presents the values of population standard deviation (according to Formula (1)) specified for the values of strains obtained for particular mixes.

(1) $$s=\sqrt{\frac{\sum_{i=1}^{n}\left(x_{i}-\bar{x}\right)}{n}}$$

The population standard deviation values obtained for the tested mixtures indicate that the additions of SRW up to 5% do not significantly affect the scatter of the obtained results. Only the addition of 10% SRW causes significant decreases in the standard deviation values. These values indicate the influence of SRW additives on the uniformity of stress distribution and, as a result, strain on the surface of the tested samples.

Note also the strain values read for each mixture. In the case of values, there is a difference in the magnitude of the amplitude at each loading cycle and the magnitude of the decrease in the minimum deformation values between successive cycles. In the case of strain measurement, it can be observed that, in addition to the above-mentioned relationships, the value of strain itself decreases as the SRW content increases, but maintaining its effect on the magnitude of the amplitude. It is assumed that the maximum values of strain readings should oscillate around 1.8%, which corresponds to a deformation of 1.5 mm at an average specimen height of 80 mm, determined according to Formula (2):

(2) $$\varepsilon =\underset{L\to 0}{\lim}\frac{∆L}{L}$$

This indicates the effect of SRW on the change in the deformability of the specimen within the assumed measurement range, 70 mm with 80 mm being the height of the specimen. In addition, this is confirmed by the fact that, in the case of G0 control specimens, the maximum strain values obtained are closest to the theoretical value and only each subsequent addition causes a gradual decrease in this value.

### 3.2. Values of Elastic Strain for Individual Load Cycles

**Figure 15:**
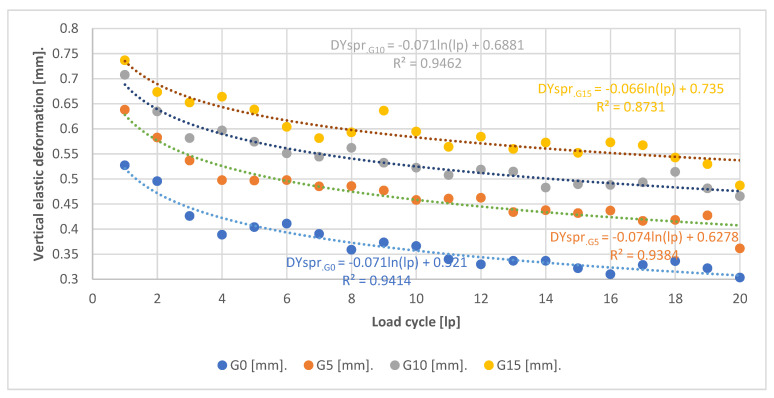
Elastic deformation values.

**Figure 16:**
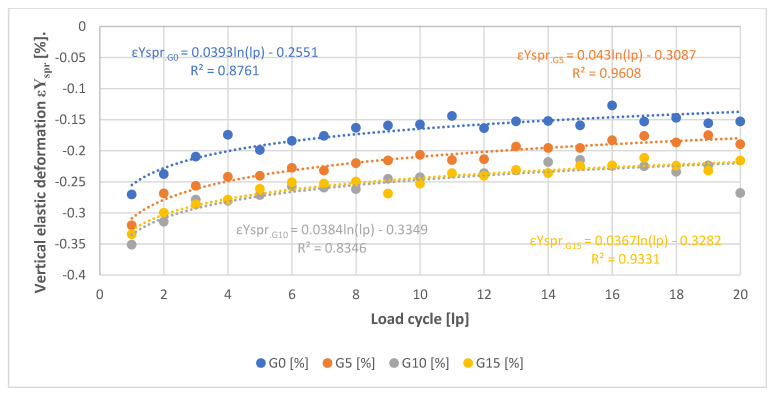
Elastic strain values.

Figure 15 and Figure 16 show plots of the values of deformation and elastic strain obtained in successive loading cycles. These values were determined from the formulas:

(3) $$DY_{spr}=DY_{max}-DY_{min}$$

where:

- *DY_spr_*—vertical elastic deformations (mm).
- *DY_max_*—vertical deformation read at maximum forcing (mm).
- *DY_min_*—vertical deformation read at minimum assumed force (mm).

(4) $$\varepsilon Y_{spr}=\varepsilon Y_{max}-\varepsilon Y_{min}$$

where:

- *εY_spr_*—vertical elastic strains [%].
- *εY_max_*—vertical strain at maximum forcing [%].
- *εY_min_*—vertical strain at minimum assumed force [%].

The graphs presented (Figure 15 and Figure 16) confirm the relationships noted in Figure 11, Figure 12, Figure 13 and Figure 14. In Figure 15, it can be seen that the SRW additives cause a uniform increase in the range of elastic deformations in which the specimen operates for each 5% of additive content. The tested mixtures obtained the following magnitudes of elastic deformations in the first and nineteenth loading cycles:

- G0—0.527 mm; 0.322 mm
- G5—0.638 mm; 0.428 mm
- G10—0.708 mm; 0.481 mm
- G15—0.736 mm; 0.529 mm

The magnification of the elastic operation range is similar for the first and last test cycle. It can also be seen that, in the case of G0 control specimens, there is a sharp reduction in the magnitude of elastic deformation in the first load cycles, whereas for G5, G10 and G15 specimens this reduction is more gentle. Specimens G5 and G10 also exhibit the smallest reduction in elastic deformation range between the first and last loading cycles. In the case of specimens G0 and G15, the values are similar.

From Figure 16, it can be seen that SRW additions less than or equal to 10% cause an increase in the elastic strain range. In the case of the 15% addition, it presents values very close to the 10% addition. The trend of the effect of the SRW content on the strain values is very similar to that observed for global deformation measurements. However, the most significant difference is observed in the magnitude of strain values. These values are about three times lower than those that can be calculated using equation 2, based on the elastic deformation values from Figure 15.

### 3.3. Map of Vertical Strains Occurring on the Specimen Surface

**Figure 17:**
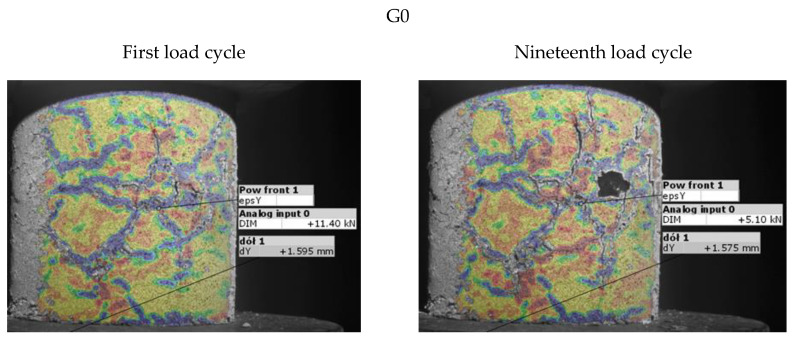

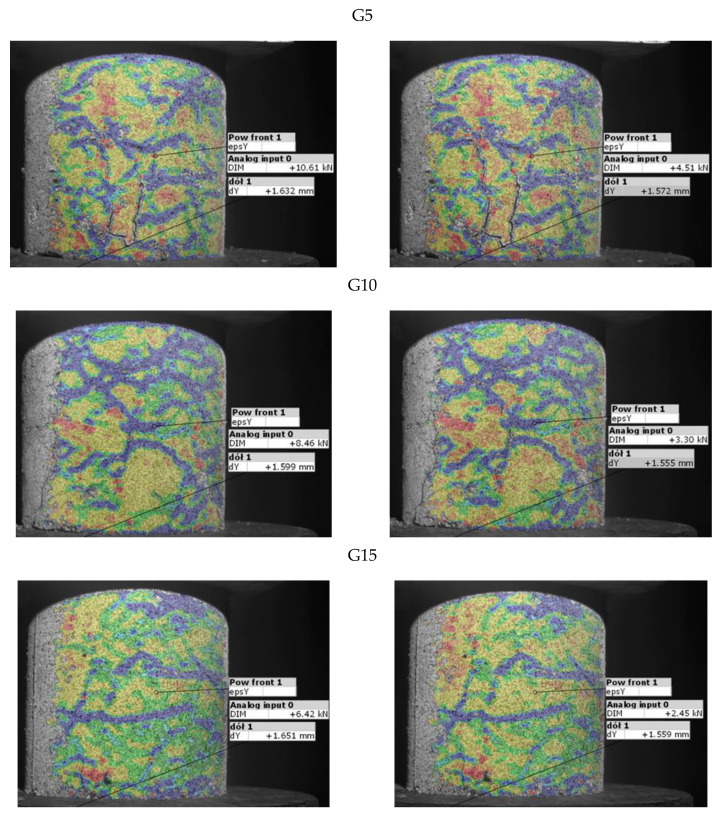
Maps of vertical strains in the first and nineteenth loading cycle for selected specimens.

**Figure 18:**
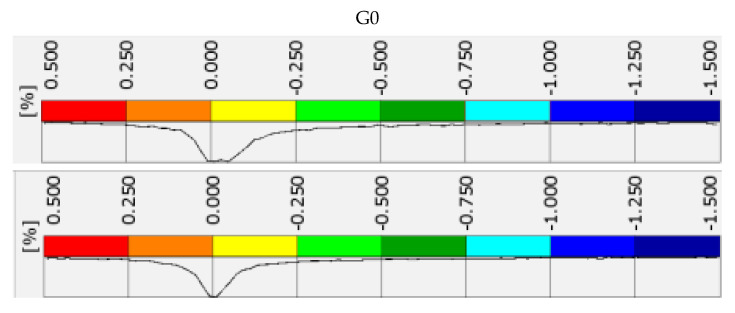

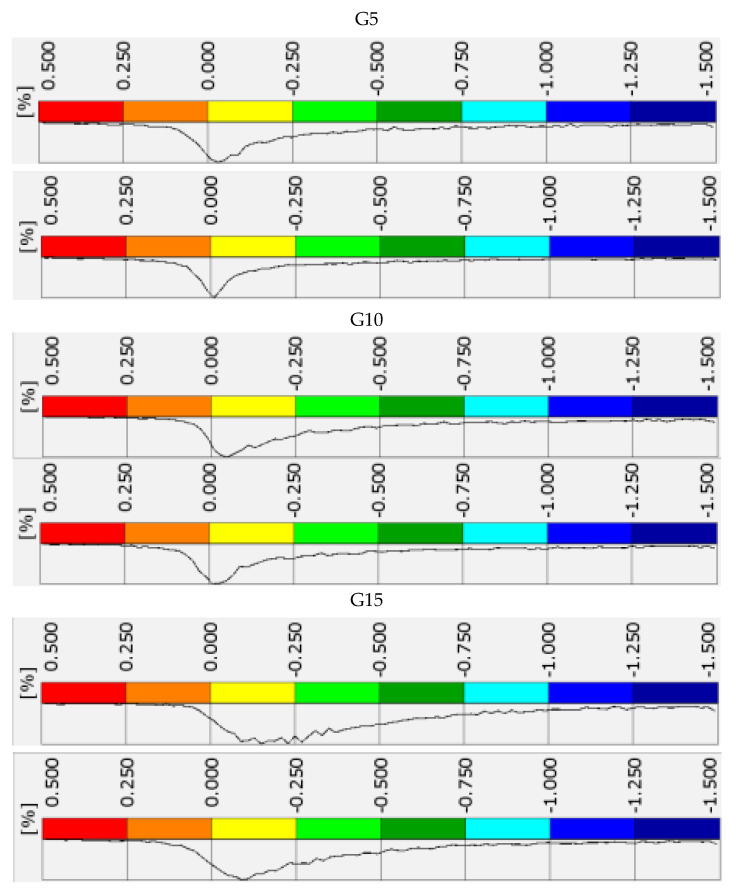
Histograms of vertical strain distribution in the first and nineteenth loading cycle at maximum deformation forcing for selected specimens shown in Figure 17.

Figure 17 shows the vertical strain maps, at the specimen surface, at the maximum forcing in the first and nineteenth loading cycles. The images show one selected specimen from each mixture. Attached to the strain maps are histograms of the distribution of strain values on the specimen surface, along with a legend, included in Figure 18.

As can be seen in Figure 17, SRW additions greater than 5% allow the specimens to work in a larger deformation range (1.5 mm; ε = 1.9%) without developing clear scratches. In the case of specimens G0 and G5, clear scratches can be seen already in the first loading cycle, which develop until the nineteenth cycle. In the case of specimens G10 and G15, such large scratches do not occur.

It is also important to note the effect of SRW content on the strain distribution on the specimen surface. In the case of G0 control specimens, we are dealing with large areas of strain between 0 and 0.25%, interspersed with high strain (greater than or equal to 1.5%) scratches. As the SRW content increases, we can observe a gradual decrease in areas with very low strains, which are replaced by fields with strains up to 0.5%. This phenomenon is perfectly visible in Figure 18, by the shift of the peak of the strain distribution towards negative values. In the case of specimen G15, we now see predominantly areas of strain up to 0.5%, interspersed with areas of high strain accumulated in the lower and upper regions of the specimen.

## 4. Discussion

The research results presented in the paper indicate the influence of SRW content on the distribution of deformations in the sample caused by the applied load. This resulted in an increase in the range of elastic strains along with an increase in SRW content in the sample, despite the presence of rigid hydraulic bonds in them. Increasing the elasticity of the composite should allow for the improvement of its fatigue life, which is the most important aspect of road pavement due to the load characteristics that occur on it. Calculations of the values of standard deviations of the population of vertical strains measurements indicate an improvement in the uniformity of deformation of the mixture with an increase in the content of SRW. This, in turn, allows us to state that, in the case of using SRW additives, local phenomena occurring in the structure of the material for the benefit of its global work decreases. It is particularly important when using unburnt coal-mining slate as aggregate constituting the skeleton of the mixture, because its load-bearing properties are very dependent on the place of its collection and the time remaining in the heap due to erosive processes taking place at that time. Such behaviour of the composite caused by SRW additives may allow for easier and more convenient use of mining waste in the construction of the road pavement. The effect of the use of SRW additives on the improvement of the homogeneity of composite deformation under an applied load is also noticeable when observing the deformation maps of samples. It is visible there that as SRW content increases, less scratches with high vertical deformation values appear in favour of more extensive fields with lower deformation values. Additionally, it can be noticed that along with the discussed homogenization of the deformation distribution, the samples with the highest SRW content do not show significant damage to their surface despite the occurrence of plastic deformations in them and despite the very high deformation threshold that was set in the test. This deformation threshold significantly exceeds the deformation values that can be noted in the layers of the pavement structure. This again indicates that this type of composite can better withstand fatigue conditions. The material with the presented characteristics, combined with the influence of SRW content on the reduction of the mass water absorption value and the water capillary rise, presented in the earlier works of the authors [55,56], may be an interesting alternative to standard materials used in the road construction. Especially as mainly waste materials are used, it fits very well with the trend towards sustainable ecological construction. However, in order to be able to safely use it in construction, more tests, frost resistance in particular, and test loading of the model of bituminous layer systems placed on the foundation made of SRFC composite should be carried out. The tests of the models are to show whether the uniform deformability and the increased range of elastic deformations have a positive or negative effect on the functional properties of the road pavement constructed in this way.

## 5. Conclusions

**Figure 19:**
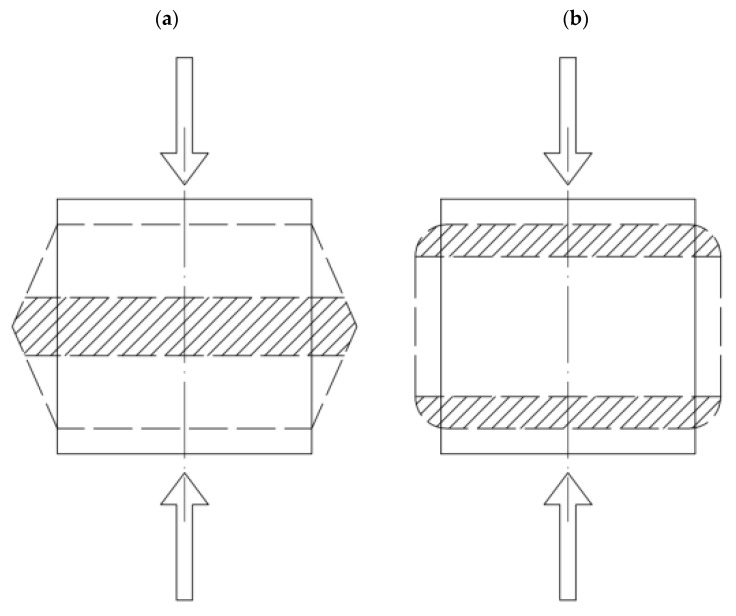
Form of strain: (**a**) rigid specimen; (**b**) specimen with high content of shredded rubber waste.

Based on the above results, it can be concluded that SRW additives cause:

- Enlargement of the deformation range in which the specimen works elastically
- Decreasing tendency to reduce the elastic deformation range with each successive load cycle
- Alignment of the distribution of strains on the specimen surface
- Change in the strain form of the specimen
- The differences in the values of strains measured with virtual strain gauges and global deformations are due to the change in the form of strain of the specimen caused by the application of SRW additives. In the case of G0 control specimens, we are dealing with the form of deformability similar to that presented in Figure 19a, for which the strains are transmitted through the rigid parts of the specimen along the loading axis. In this case, the failure of the specimen occurs as a result of the accumulation of strains in the central part of the specimen, whereby the exhaustion of the load carrying capacity occurs. Specimens with SRW additives have lower stiffness, so that strains are not immediately transmitted along the load axis. They accumulate in the contact zones of the specimen and are only transmitted further when their local deformability is exhausted (Figure 19b). Therefore, the strain gauges, which did not cover the entire height of the specimen, did not record the total strain, which was realized in the contact zones of the specimen with the testing machine attachments.

As a result of this profile, the specimen is less sensitive to the effects of local phenomena resulting from, e.g., the arrangement of aggregate grains in the mixture, and shows a higher deformability under applied load. Additionally, specimens containing SRW additives under less-than-destructive loading will be characterized by the occurrence of two zones:

- A buffer zone located where the load is in contact with the specimen and where the specimen is supported. This zone will absorb a significant portion of the strain
- Core zones where the inner part of the specimen will be subject to slight strain.

## Figures and Tables

**Table 1:** Proctor Methods.

Method	Mass of Rammer (kg)	Volume of Mold (dm^3^)	Compaction Energy (kN × m^−1^ × dm^−3^)
I	2.5	1.0	0.59
II	2.5	2.2	0.59
III	4.5	1.0	2.65
IV	4.5	2.2	2.65

**Table 2:** Percentage of additives determined in relation to dry weight of unburnt coal-mining slate.

Recipe	Shredded Rubber Waste (%)	Fly Ah (%)	Cement CEM I 42.5R (%)
G0	0	5	5
G5	5	5	5
G10	10	5	5
G15	15	5	5

**Table 3:** Population standard deviation values for strain measurements averaged over the mixtures tested.

Mix	Population Standard Deviation [%]
G0	0.344
G5	0.333
G10	0.226
G15	0.219

## Data Availability

Not applicable.
